# Health effects related to exposure of static magnetic fields and acoustic noise—comparison between MR and CT radiographers

**DOI:** 10.1007/s00330-022-08843-y

**Published:** 2022-06-08

**Authors:** Anton Glans, Jonna Wilén, Lenita Lindgren, Isabella M. Björkman-Burtscher, Boel Hansson

**Affiliations:** 1 Department of Nursing, Umeå University, Umeå, Sweden; 2 Department of Radiation Sciences, Radiation Physics, Umeå University, Umeå, Sweden; 3 Department of Radiology, Institute of Clinical Sciences, Sahlgrenska Academy, University of Gothenburg, Gothenburg, Sweden; 4 Department of Radiology, Sahlgrenska University Hospital, Västra Götalands Region, Gothenburg, Sweden; 5 Department of Medical Imaging and Physiology, Skåne University Hospital, Lund, Sweden; 6 Department of Diagnostic Radiology, Clinical Sciences, Lund University, Lund, Sweden

**Keywords:** Magnetic resonance imaging, Occupational health, Tomography, X-ray computed, Surveys and questionnaires, Electromagnetic fields

## Abstract

**Objectives:**

We explored the prevalence of health complaints subjectively associated with static magnetic field (SMF) and acoustic noise exposure among MR radiographers in Sweden, using CT radiographers as a control group. Additionally, we explored radiographers’ use of strategies to mitigate adverse health effects.

**Methods:**

A cross-sectional survey was sent to all hospitals with MR units in Sweden. MR and/or CT personnel reported prevalence and attribution of symptoms (vertigo/dizziness, nausea, metallic taste, illusion of movement, ringing sensations/tinnitus, headache, unusual drowsiness/tiredness, forgetfulness, difficulties concentrating, and difficulties sleeping) within the last year. We used logistic regression to test associations between sex, age, stress, SMF strength, working hours, and symptom prevalence. Data regarding hearing function, work-environmental noise, and strategies to mitigate adverse symptoms were also analysed.

**Results:**

In total, 529 out of 546 respondents from 86 hospitals were eligible for participation. A ≥ 20 working hours/week/modality cut-off rendered 342 participants grouped into CT (*n* = 75), MR (*n* = 121), or mixed personnel (*n* = 146). No significant differences in symptom prevalence were seen between groups. Working at ≥ 3T increased SMF-associated symptoms as compared with working at ≤ 1.5T (OR: 2.03, CI_95_: 1.05–3.93). Stress was a significant confounder. Work-related noise was rated as more troublesome by CT than MR personnel (*p* < 0.01). MR personnel tended to use more strategies to mitigate adverse symptoms.

**Conclusion:**

No significant differences in symptom prevalence were seen between MR and CT radiographers. However, working at 3T increased the risk of SMF symptoms, and stress increased adverse health effects. Noise nuisance was considered more problematic by CT than MR personnel.

**Key Points:**

• *No significant differences in symptom prevalence were seen between MR and CT radiographers.*

• *Working at ≥ 3 T doubled the odds of experiencing SMF symptoms (vertigo/dizziness, nausea, metallic taste, and/or illusion of movement) as compared to working exclusively at ≤ 1.5 T.*

• *Work-related acoustic noise was less well mitigated and was rated as more troublesome by CT personnel than by MR personnel.*

## Introduction

Working with MRI entails static magnetic field (SMF) exposures that are thousands of times stronger than the Earth’s magnetic field. The exposure is expressed as magnetic flux density measured in Tesla (T), and clinical field strengths commonly range from 1 to 7 T [1, 2]. Any physical movement inside the SMF creates motion-induced time-varying magnetic fields in the body, which induce electrical currents that can stimulate sensory organs [3, 4]. MR personnel, e.g., radiographers, move inside the SMF as an integrated part of their work: setting up equipment, assisting and positioning patients, and cleaning between exams. In some instances, MR personnel might be needed inside the room during scanning for patient support. Furthermore, switched gradient magnetic fields are applied during scanning which produce acoustic noise between 80 and 130 decibels (dB) [5, 6]—sound pressure levels that can cause discomfort and hearing injuries (≥ 85 dB) [5, 7].

Exposure to any one of the above sources is a potential health hazard. MR personnel report transient symptoms such as vertigo, tiredness, metallic taste, headache, nystagmus, head ringing, and nausea [3, 8, 9]. Some workers also experience visual light flashes (magnetophosphenes) and have difficulties concentrating [10–12]. Many of the transient symptoms are linked to SMF exposure [13], particularly during movement of the head. Recent evidence suggests that the magnetic fields induce electric fields, which affect the vestibular system and initiate vertigo [14, 15], especially at stronger SMFs, e.g., 7 T [16, 17].

To mitigate adverse health effects from SMF exposure, occupational exposure limits are set by the International Commission on Non-Ionizing Radiation Protection (ICNIRP) [18]. These limits form a basis for national legislation—for instance, AFS 2016:3 [19] in Sweden. Moreover, personnel are recommended to use hearing protection inside the MR room during scanning [7, 20], and to move slowly close to the MR bore to limit motion-induced symptoms [11, 21].

The discussion about safety in MR environments intensified after the first draft of the EU directive 2013/35/EU [22] was published in 2009. At the same time, a Swedish pilot study among MR personnel found that 47% reported symptoms such as dizziness, illusion of movement, and headaches regularly, and 15% experienced symptoms in connection to their routine work close to the scanner [8]. However, a limitation in that and similar studies [13, 23] was the lack of control groups to account for environmental confounders. To limit adverse health effects, we must better understand the factors in the working environment that contribute to causing harm [4]. We propose to adjust for work-related confounders by comparing health effects among MR personnel with a similar group working without exposure to strong SMFs—i.e., CT personnel. Incidentally, established work environmental risk exposures among CT personnel outside of ionizing radiation are limited; however, stress and burnout appear to be increasing among CT radiographers due to increased workload [24].

Our primary aim was to explore the prevalence of health complaints subjectively associated with static magnetic field and acoustic noise exposure among MR radiographers in Sweden, using CT radiographers as a control group. Our main hypothesis was that MR radiographers reported more symptoms in general compared to CT radiographers. Additionally, we explored the attribution of symptoms in relation to modalities and whether personnel use strategies to mitigate adverse health effects.

## Material and methods

Data to assess health complaints were extracted from a cross-sectional national survey with 202 items that had been sent electronically to the 92 known hospitals with MR units in Sweden. At each hospital, a contact person responsible for MRI and/or CT was asked to inform their colleagues and distribute a link to the web-based questionnaire. Data from the same questionnaire, focusing on MR incidents, have earlier been published and we refer to this publication for further information on data collection procedures [25]. In summary, all personnel working to any degree with MRI and/or CT scanning—thus, primarily targeting radiographers—were considered eligible for participation. At the time, the number of personnel working with human scanning within the 92 hospitals was estimated to be 620 for MR and approximately 1300 for CT [25]. Data were collected between September 2015 and April 2016, during which time three survey reminders were sent to increase the response rate.

The included items were based on similar validated questionnaires concerning work environmental health, e.g., the widely used MM-questionnaires, and The Occupational Stress Questionnaire [26, 27]. Specifically, participants were asked to rate the prevalence of symptoms subjectively associated with SMF and acoustic noise exposure: vertigo/dizziness, nausea, metallic taste, illusion of movement, ringing sensation or sound (tinnitus), headache, unusual drowsiness or tiredness, forgetfulness, difficulties concentrating, and difficulties sleeping. We asked for symptom prevalence in general during the last year, e.g., without taking their work with MR or CT into account. Prevalence ranged on a five-point Likert scale from “*never*” to “*more than four times a week*”. This was followed by questions about whether symptoms occurred or intensified when being in the MR and/or CT scanner room, and if so, in relation to where in the room and doing what type of movements, if any. These items had previously been tested among MR nurses in a pilot study [8]. Besides demographic data, participants were also asked about work-environmental noise, self-rated hearing function, and if they used any strategies to mitigate adverse health effects in their work.

First, we included all eligible participants and used their amount of weekly MR working hours as an independent variable to predict symptom prevalence. Second, we categorized participants into three separate groups: *MR personnel*, *CT personnel*, and *mixed* (both MR and CT) *personnel*. To meet group inclusion, we added a cut-off value *a priori* of 20 work hours/week (h/w), i.e., ≥ 50% of full-time work in Sweden, within the respective image modalities. Personnel who worked ≥ 20 h/w with MR, and did not work with CT at all, were categorized into the MR group. Personnel who worked ≥ 20 h/w with CT, and not with MR at all, were categorized into the CT group. The mixed group consisted of participants who worked both with CT and MR for at least 20 h/w combined. Third, we divided all MR users from *MR personnel* and *mixed personnel* into those whose work included MR systems > 1.5 T and those working only at ≤ 1.5 T.

All symptom prevalence variables were *a priori* dichotomized into *never*/*seldom* (< 1 time/week) or *often* (≥ 1 time(s)/week), as previously done by Wilén and de Vocht [8]. The symptoms were also categorized into three groups in relation to *SMF* (vertigo/dizziness, illusion of movement, metallic taste, and nausea), *general work environment* (difficulties concentrating, difficulties sleeping, forgetfulness, headache, and unusual drowsiness or tiredness), and *acoustic noise* (ringing sensations or sound/tinnitus). The variable “are you experiencing acoustic noise in your work in general as troublesome?” was dichotomized into *little* (“not at all” or “somewhat”) or *a great deal*. Additionally, the following variables were dichotomized: sex; established hearing loss (yes/no), and self-rated hearing function (normal/impaired).

We used descriptive statistics to survey symptom prevalence and display participant characteristics. When appropriate, we used the Chi-square tests to compare categorical data between the three groups. Logistic regression was used to test the association between MR work exposure (h/w) and symptom prevalence, which included all eligible participants. Moreover, we tested the association between symptom prevalence and *MR*, *CT*, and *mixed* personnel. Symptoms were both tested individually and within the three symptom groups as dependent variables in separate logistic regression models. Lastly, MR users within the MR and mixed personnel were analyzed to evaluate whether SMF strength affected the prevalence of the three symptom groups. In all logistic regression models, adjustment variables were age, sex, and stress level. Each independent variable was initially tested using univariate logistic regression. Any variable with a *p* value < 0.2 was included in a multivariate regression model to adjust for confounders. Missing data in any of the logistic regression variables were excluded from that model.

All data analyses were carried out in SPSS version 26 (IBM Corp.). In all tests, a *p* value ≤ 0.05 was considered statistically significant.

## Results

### Participants

Our survey was answered by 546 participants from 86 hospitals. After we excluded incomplete submissions and subjects who neither worked with MR, CT, nor human scanning, 529 participants were eligible, among which 345 worked with MR, and 392 worked with CT. Adding the cut-off value of 20 work h/w within the image modalities rendered 342 participants for group inclusion. Among these, 121 participants worked ≥ 50% with MR but not CT (78% female; mean age ± SD: 52 ± 8.9 years, range 30–66), 75 participants worked ≥ 50% with CT but not MR (84 % female; mean age ± SD: 43 ± 13 years, range 23–65), and 146 participants worked ≥ 50% with both MR and CT combined (71% female; mean age ± SD 42 ± 12 years, range 24–65). Except for two biomedical scientists, one registered nurse, and one physicist, all participants were radiographers. Demographic data are presented in Table 1. Altogether, 267 (121 + 146) MR personnel were included in the cut-off stratification.

**Table 1:** Demographics (*n* = 342)

		CT (n = 75)	MR (n = 121)	Mixed (n = 146)	
Sex, n (%)	Male	12 (16%)	27 (22%)	43 (29%)	
	Female	63 (84%)	94 (78%)	103 (71%)	
Age, mean ± SD; range		42 ± 13; 23–65	52 ± 8.9; 30–66	42 ± 12; 24–65	
Occupation, n (%)	Radiographer	73 (97%)	119 (98%)	146 (100%)	
	Other	2 ^a^ (2.7%)	2 ^b^ (1.7%)	0 (0%)	
Stress	Not at all (1), n (%)	14 (19%)	33 (27%)	26 (18%)	
	Only a little (2), n (%)	22 (29%)	26 (22%)	43 (30%)	
	To some extent (3), n (%)	15 (20%)	38 (32%)	43 (30%)	
	Rather much (4), n (%)	19 (25%)	17 (14%)	25 (18%)	
	Very much (5), n (%)	5 (6.7%)	6 (5.0%)	5 (3.5%)	
	Missing^c^, n (%)	0	1 (0.8%)	5 (3.4%)	
Field strength, B_0_, (multiple choice), n (%)	1T	---	2 (1.7%)	6 (4.1%)	
	1.5 T	---	115 (95%)	142 (97%)	
	3T	---	60 (50%)	38 (26%)	
	7T	---	1 (0.8%)	0 (0%)	
Perceived health state	Good, n (%)	44 (59%)	61 (51%)	87 (61%)	
	Average, n (%)	29 (39%)	56 (47%)	53 (37%)	
	Not so good, n (%)	2 (2.7%)	3 (2.5%)	3 (2.1%)	
	Missing^c^, n (%)	0	1 (0.8%)	3 (2.1%)	
Sick leave days within last year	0 days, n (%)	26 (35%)	49 (41%)	49 (35%)	
	≤ 9 days, n (%)	38 (51%)	55 (46%)	74 (53%)	
	10–24 days, n (%)	8 (11%)	9 (7.5%)	13 (9.3%)	
	25–99 days, n (%)	3 (4%)	5 (4.2%)	4 (2.9%)	
	100–365 days, n (%)	0	2 (1.7%)	0	
	Missing^c^, n (%)	0	1 (0.8%)	6 (4.1%)	
Working hours (multiple choice), n (%)	Daytime (~7.00–17.00), n (%)	74 (99%)	120 (99%)	146 (100%)	
	Evening (~17.00–21.00), n (%)	44 (59%)	62 (51%)	122 (84%)	
	Weekends, n (%)	45 (60%)	35 (29%)	112 (77%)	
	Night (~21.00–7.00), n (%)	23 (31%)	1 (0.8%)	70 (48%)	
	On call at home, n (%)	17 (23%)	6 (5.0%)	59 (40%)	
Number of years working with CT/MR, mean ± SD; range				**At CT**	**At MR**
		11 ± 9.0; 1–37	14 ± 6.9; 2–32	13 ± 9.4; 2–36	8.3 ± 6.2; 1–30
Estimated time (h/workday) in the CT/MR room	0 h, n (%)	3 (4.1%)	1 (0.8%)	2 (1.5%)	1 (0.7%)
	> 0 - ½ h, n (%)	6 (8.1%)	2 (1.7%)	9 (6.6%)	10 (6.9%)
	> ½ - 1 h, n (%)	5 (6.8%)	26 (22%)	22 (16%)	55 (38%)
	> 1 - 1½ h, n (%)	10 (13%)	52 (44%)	31 (23%)	41 (28%)
	> 1½ - 2h, n (%)	14 (19%)	28 (24%)	30 (22%)	25 (17%)
	> 2 h, n (%)	36 (49%)	9 (7.6%)	43 (31%)	13 (9.0%)
	Missing^c^, n (%)	1 (1.3%)	3 (2.5%)	9 (12%)	1 (0.7%)

*CT*, participants working ≥ 20 hours/week (h/w) with CT and not MR; *MR*, participants working ≥ 20 h/w with MR and not CT; *Mixed*, participants working ≥ 20 h/w with both CT and MR combined; other^a^, one biomedical scientist and one registered nurse; other^b^, one biomedical scientist and one physicist. ^c^Missing data are accounted for by calculating % based on the number of responses for each item—except for the multiple-choice items “working hours” and “field strength” where participants could choose one or multiple alternatives for each question and % for each alternative is based on the maximum individual participants in each group (CT: 75; MR: 121; mixed: 146)

### Health complaints

Reported symptom prevalence (*n* = 342 participants) did not differ significantly between the MR, CT, and mixed groups (all *p* > 0.05) (Table 2). Among participants in the MR and mixed group (*n* = 267), however, working with ≥ 3 T doubled the odds of experiencing SMF symptoms ≥ 1 time(s)/week (odds ratio, OR: 2.03; CI_95_: 1.05–3.93, *p* = 0.04).

**Table 2:** Symptom prevalence in general (*n* = 342) and logistic regressions including SMF strength association with symptom groups (*n* = 267)

Symptom groups	Prevalence	CT (n = 75)	MR (n = 121)	Mixed (n = 146)	*p*-value (crude/adjusted)	Static magnetic field strength		
						Work only includes ≤ 1.5 T (n = 169)	Work includes ≥ 3 T (n = 98)	
**Static magnetic field**						**Experiencing static magnetic field symptoms ≥ 1 time(s) a week**		
Vertigo/dizziness, n (%)	*Never - seldom*	69 (95%)	108 (92%)	129 (92%)	0.74/0.62	Variable	OR (95% CI)	*p*-value
	*≥ 1 time(s)/week*	4 (5.5%)	10 (8.5%)	11 (7.9%)		Work includes 3 T	2.03 (1.05 – 3.93)	**0.04**
Nausea, n (%)	*Never - seldom*	70 (96%)	113 (95%)	133 (95%)	0.95/0.96	Stress [not at all]		**0.01**
	*≥ 1 time(s)/week*	3 (4.1%)	6 (5.0%)	7 (5.0%)		Only a little	2.31 (0.78 – 6.85)	0.13
Metallic taste, n (%)	*Never - seldom*	72 (100%)	116 (98%)	139 (99%)	0.78/0.95	To some extent	2.37 (0.81– 6.97)	0.12
	*≥ 1 time(s)/week*	0 (0%)	2 (1.7%)	1 (0.7%)		Rather much	2.07 (0.64 – 6.76)	0.23
Illusion of movement, n (%)	*Never - seldom*	72 (99%)	116 (97%)	131 (95%)	0.33/0.09	Very much	11.9 (3.04 – 46.3)	< 0.01
	*≥ 1 time(s)/week*	1 (1.4%)	3 (2.5%)	7 (5.1%)		**Experiencing general work environment symptoms ≥ 1 time(s) a week**		
**General work environment**						Variable	OR (95% CI)	*p*-value
Difficulty sleeping, n (%)	*Never - seldom*	57 (78%)	98 (82%)	111 (80%)	0.77/0.53	Work includes 3 T	0.90 (0.52 – 1.56)	0.70
	*≥ 1 time(s)/week*	16 (22%)	21 (18%)	27 (20%)		Stress [not at all]		**< 0.01**
Fatigue/drowsiness, n (%)	*Never - seldom*	64 (88%)	104 (87%)	108 (78%)	0.11/0.08	Only a little	1.97 (0.86 – 4.49)	0.11
	*≥ 1 time(s)/week*	9 (12%)	16 (13%)	30 (22%)		To some extent	5.70 (2.60 – 12.5)	**< 0.01**
Difficulty concentrating, n (%)	*Never - seldom*	65 (89%)	101 (84%)	121 (87%)	0.61/0.34	Rather much	12.0 (5.03 – 28.7)	**< 0.01**
	*≥ 1 time(s)/week*	8 (11%)	19 (16%)	18 (13%)		Very much	13.3 (3.80 – 46.4)	**< 0.01**
Amnesia/forgetfulness, n (%)	*Never - seldom*	69 (95%)	112 (93%)	126 (91%)	0.66/0.43	**Experiencing acoustic noise symptoms ≥ 1 time(s) a week**		
	*≥ 1 time(s)/week*	4 (5.5%)	8 (6.7%)	12 (8.7%)		Variable	OR (95% CI)	*p*-value
Headache, n (%)	*Never - seldom*	63 (86%)	105 (89%)	122 (88%)	0.85/0.91	Work includes 3 T	1.24 (0.54 – 2.85)	0.62
	*≥ 1 time(s)/week*	10 (14%)	13 (11%)	16 (12%)		Age	1.04 (1.00–1.08)	0.05
**Acoustic noise**						Stress [not at all]		0.11
Tinnitus/ringing sound, n (%)	*Never - seldom*	65 (89%)	113 (94%)	127 (91%)	0.42/0.23	Only a little	3.92 (0.80 – 19.4)	0.09
						To some extent	3.00 (0.60 – 15.1)	0.18
	*≥ 1 time(s)/week*	8 (11%)	7 (5.8%)	13 (9.3%)		Rather much	7.53 (1.57 – 36.3)	**0.01**
						Very much	2.61 (0.22 – 31.2)	0.45

Prevalence data are presented in counts and proportions (%). Missing data are accounted for by calculating % based on the number of responses for each symptom. We used logistic regression to test if any symptom prevalence was associated with any specific personnel group (crude), and also adjusted the models for age, sex, and stress level (adjusted)

To compare each symptom group’s prevalence in relation to static magnetic field strengths (SMFs), we divided all MR-working participants (both MR and mixed, *n* = 267) into those whose work only included ≤ 1.5 T (*n* = 169) and those whose work included ≥ 3 T (*n* = 98), and tested with logistic regression adjusted for sex, age, and stress (ascending order); “Work only includes ≤ 1.5 T”, and “not at all” stress level was chosen as the reference categories. To simplify each SMF model, we excluded all variables with a *p* value > 0.2. *OR*, odds ratio; *CI*, confidence interval

**Fig. 1:**
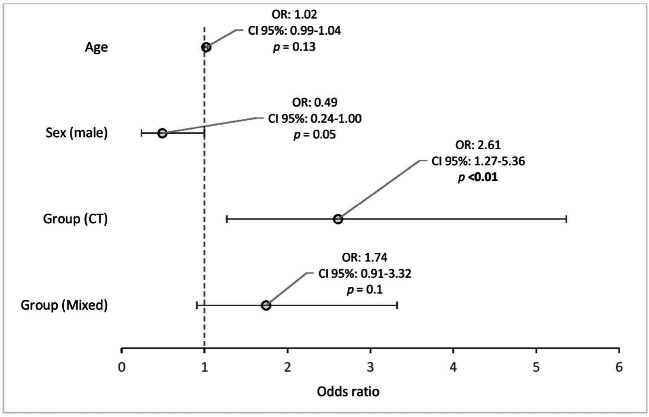
Odds ratios of experiencing acoustic noise at work in general as troublesome to “a great deal” (*n* = 342). We dichotomized the outcome of “are you experiencing acoustic noise at work in general as troublesome?” into 0 = “*not at all*” or “*somewhat*”, and 1 = “*a great deal”*, and performed logistic regression adjusted for sex, age, and stress (ascending order). To simplify the model, we excluded all variables with a *p* value > 0.2. Female sex, and group (MR), were the reference categories. Plots denote the odds ratio, OR, and the black whiskers mark the 95% confidence interval (CI)

The CT group showed 2.6 times greater odds of experiencing acoustic noise at work in general as troublesome to “a great deal” than the MR group (*p* < 0.01) (Fig. 1). Occasional ringing sounds were reported to occur when inside the CT room or standing next to the gantry (Tables 3 and 4). However, no differences were seen in self-rated hearing function (*p* = 0.54) or established hearing loss (*p* = 0.79) between CT, MR, or mixed personnel (Table 5).

**Table 3:** Symptom occurrence (*n* = 342)

Symptom, n (%)		CT (n = 75)	MR (n = 121)	Mixed (n = 146)	
				At CT	At MR
**Static magnetic field**					
Vertigo/dizziness	Never occurs	58 (77%)	55 (45%)	113 (77%)	76 (52%)
	Sometimes occurs	12 (16%)	59 (49%)	11 (7.5%)	52 (36%)
	Always occurs	0 (0%)	1 (0.8%)	0 (0%)	4 (2.7%)
	Occurs immediately when entering the CT/MR room	0 (0%)	1 (0.8%)	0 (0%)	2 (1.4%)
	Occurs after being in the CT/MR room for a while	0 (0%)	7 (5.8%)	1 (0.7%)	9 (6.2%)
	Remains after leaving the CT/MR room	0 (0%)	1 (0.8%)	1 (0.7%)	1 (0.7%)
Nausea	Never occurs	62 (83%)	100 (83%)	116 (79%)	126 (86%)
	Sometimes occurs	6 (8.0%)	14 (12%)	8 (5.5%)	11 (7.5%)
	Always occurs	0 (0%)	0 (0%)	0 (0%)	0 (0%)
	Occurs immediately when entering the CT/MR room	0 (0%)	0 (0%)	0 (0%)	1 (0.7%)
	Occurs after being in the CT/MR room for a while	0 (0%)	4 (3.3%)	0 (0%)	1 (0.7%)
	Remains after leaving the CT/MR room	0 (0%)	3 (2.5%)	1 (0.7%)	1 (0.7%)
Metallic taste	Never occurs	69 (92%)	103 (85%)	123 (84%)	135 (92%)
	Sometimes occurs	0 (0%)	8 (6.6%)	1 (0.7%)	1 (0.7%)
	Always occurs	0 (0%)	0 (0%)	0 (0%)	0 (0%)
	Occurs immediately when entering the CT/MR room	0 (0%)	0 (0%)	0 (0%)	0 (0%)
	Occurs after being in the CT/MR room for a while	0 (0%)	1 (0.8%)	0 (0%)	0 (0%)
	Remains after leaving the CT/MR room	0 (0%)	1 (0.8%)	1 (0.7%)	1 (0.7%)
Illusion of movement	Never occurs	62 (83%)	85 (70%)	117 (80%)	107 (73%)
	Sometimes occurs	6 (8.0%)	29 (24%)	7 (4.8%)	26 (18%)
	Always occurs	0 (0%)	1 (0.8%)	0 (0%)	1 (0.7%)
	Occurs immediately when entering the CT/MR room	1 (1.3%)	1 (0.8%)	0 (0%)	1 (0.7%)
	Occurs after being in the CT/MR room for a while	0 (0%)	5 (4.1%)	2 (1.4%)	6 (4.1%)
	Remains after leaving the CT/MR room	0 (0%)	2 (1.7%)	0 (0%)	2 (1.4%)
**General work environment**					
Difficulty concentrating	Never occurs	50 (67%)	97 (80%)	107 (73%)	111 (76%)
	Sometimes occurs	19 (25%)	17 (14%)	16 (11%)	22 (15%)
	Always occurs	0 (0%)	0 (0%)	0 (0%)	1 (0.7%)
	Occurs immediately when entering the CT/MR room	0 (0%)	1 (0.8%)	1 (0.7%)	0 (0%)
	Occurs after being in the CT/MR room for a while	2 (2.7%)	1 (0.8%)	2 (1.4%)	1 (0.7%)
	Remains after leaving the CT/MR room	2 (2.7%)	2 (1.7%)	3 (2.1%)	2 (1.4%)
Amnesia/forgetfulness	Never occurs	64 (85%)	102 (84%)	119 (82%)	128 (88%)
	Sometimes occurs	5 (6.7%)	12 (9.9%)	5 (3.4%)	9 (6.2%)
	Always occurs	0 (0%)	0 (0%)	0 (0%)	0 (0%)
	Occurs immediately when entering the CT/MR room	0 (0%)	0 (0%)	0 (0%)	0 (0%)
	Occurs after being in the CT/MR room for a while	0 (0%)	1 (0.8%)	0 (0%)	1 (0.7%)
	Remains after leaving the CT/MR room	0 (0%)	2 (1.7%)	0 (0%)	1 (0.7%)
Fatigue/drowsiness	Never occurs	53 (71%)	94 (78%)	106 (73%)	109 (75%)
	Sometimes occurs	13 (17%)	19 (16%)	15 (10%)	23 (16%)
	Always occurs	0 (0%)	1 (0.8%)	2 (1.4%)	1 (0.7%)
	Occurs immediately when entering the CT/MR room	0 (0%)	0 (0%)	0 (0%)	0 (0%)
	Occurs after being in the CT/MR room for a while	2 (2.7%)	2 (1.7%)	1 (0.7%)	1 (0.7%)
	Remains after leaving the CT/MR room	3 (4.0%)	1 (0.8%)	2 (1.4%)	2 (1.4%)
Headache	Never occurs	53 (71%)	94 (78%)	106 (73%)	108 (74%)
	Sometimes occurs	13 (17%)	20 (17%)	16 (11%)	26 (18%)
	Always occurs	0 (0%)	0 (0%)	1 (0.7%)	1 (0.7%)
	Occurs immediately when entering the CT/MR room	0 (0%)	0 (0%)	0 (0%)	1 (0.7%)
	Occurs after being in the CT/MR room for a while	2 (2.7%)	0 (0%)	1 (0.7%)	1 (0.7%)
	Remains after leaving the CT/MR room	4 (5.3%)	1 (0.8%)	2 (1.4%)	2 (1.4%)
**Acoustic noise**					
Tinnitus/ringing sound	Never occurs	54 (72%)	104 (86%)	114 (78%)	125 (86%)
	Sometimes occurs	11 (15%)	8 (6.6%)	6 (4.1%)	9 (6.2%)
	Always occurs	2 (2.7%)	0 (0%)	2 (1.4%)	1 (0.7%)
	Occurs immediately when entering the CT/MR room	1 (1.3%)	0 (0%)	0 (0%)	0 (0%)
	Occurs after being in the CT/MR room for a while	4 (5.3%)	0 (0%)	2 (1.4%)	0 (0%)
	Remains after leaving the CT/MR room	6 (8.0%)	2 (1.7%)	2 (1.4%)	1 (0.7%)

Participants were asked if any of the symptoms occur when working at CT or MR. All questions allowed for multiple-choice alternatives, i.e., each participant could choose none, one, or multiple alternatives for each question. Therefore, % for each alternative is based on the maximum individual participants in each group (CT: 75; MR: 121; mixed: 146). Note that the mixed group answered each statement twice—once with regards to CT and once with regards to MR

**Table 4:** Symptom attribution (*n* = 342)

Symptom	Location and movement (if any)	CT (n = 75)	MR (n = 121)	Mixed (n = 146)	
				At CT	At MR
**Static magnetic field**					
Vertigo/dizziness, n (%)	Door entrance of the MR/CT room	2 (2.7%)	2 (1.7%)	3 (2.1%)	2 (1.4%)
	Inside the MR/CT room	1 (1.3%)	4 (3.3%)	4 (2.7%)	11 (7.5%)
	Next to the gantry opening	0 (0%)	36 (30%)	1 (0.7%)	33 (23%)
	Sudden movement	13 (17%)	23 (19%)	5 (3.4%)	27 (18%)
	Turning head	4 (5.3%)	21 (17%)	1 (0.7%)	12 (8.2%)
	Leaning towards the gantry opening	0 (0%)	57 (47%)	0 (0%)	48 (33%)
Nausea, n (%)	Door entrance of the MR/CT room	1 (1.3%)	3 (2.5%)	5 (3.4%)	2 (1.4%)
	Inside the MR/CT room	3 (4%)	1 (0.8%)	4 (2.7%)	2 (1.4%)
	Next to the gantry opening	0 (0%)	8 (6.6%)	0 (0%)	4 (2.7%)
	Sudden movement	0 (0%)	2 (1.7%)	1 (0.7%)	7 (4.8%)
	Turning head	0 (0%)	3 (2.5%)	0 (0%)	3 (2.1%)
	Leaning towards the gantry opening	0 (0%)	10 (8.3%)	0 (0%)	5 (3.4%)
Metallic taste, n (%)	Door entrance of the MR/CT room	1 (1.3%)	4 (3.3%)	4 (2.7%)	3 (2.1%)
	Inside the MR/CT room	0 (0%)	0 (0%)	1 (0.7%)	0 (0%)
	Next to the gantry opening	0 (0%)	7 (5.8%)	0 (0%)	1 (0.7%)
	Sudden movement	0 (0%)	1 (0.8%)	0 (0%)	0 (0%)
	Turning head	0 (0%)	2 (1.7%)	0 (0%)	1 (0.7%)
	Leaning towards the gantry opening	0 (0%)	1 (0.8%)	0 (0%)	0 (0%)
Illusion of movement, n (%)	Door entrance of the MR/CT room	1 (1.3%)	3 (2.5%)	4 (2.7%)	3 (2.1%)
	Inside the MR/CT room	1 (1.3%)	3 (2.5%)	6 (4.1%)	4 (2.7%)
	Next to the gantry opening	1 (1.3%)	17 (14%)	0 (0%)	16 (11%)
	Sudden movement	2 (2.7%)	8 (6.6%)	2 (1.4%)	10 (6.8%)
	Turning head	2 (2.7%)	9 (7.4%)	2 (1.4%)	8 (5.5%)
	Leaning towards the gantry opening	1 (1.3%)	22 (18%)	1 (0.7%)	21 (14%)
**General work environment**					
Difficulty concentrating, n (%)	Door entrance of the MR/CT room	1 (1.3%)	3 (2.5%)	7 (4.8%)	4 (2.7%)
	Inside the MR/CT room	7 (9.3%)	6 (5.0%)	7 (4.8%)	5 (3.4%)
	Next to the gantry opening	1 (1.3%)	4 (3.3%)	0 (0%)	3 (2.1%)
	Sudden movement	1 (1.3%)	1 (0.8%)	0 (0%)	2 (1.4%)
	Turning head	0 (0%)	2 (1.7%)	0 (0%)	1 (0.7%)
	Leaning towards the gantry opening	0 (0%)	3 (2.5%)	0 (0%)	2 (1.4%)
Amnesia/forgetfulness, n (%)	Door entrance of the MR/CT room	2 (2.7%)	3 (2.5%)	6 (4.1%)	4 (2.7%)
	Inside the MR/CT room	4 (5.3%)	1 (0.8%)	3 (2.1%)	3 (2.1%)
	Next to the gantry opening	0 (0%)	1 (0.8%)	0 (0%)	2 (1.4%)
	Sudden movement	0 (0%)	0 (0%)	0 (0%)	2 (1.4%)
	Turning head	0 (0%)	0 (0%)	0 (0%)	1 (0.7%)
	Leaning towards the gantry opening	0 (0%)	1 (0.8%)	0 (0%)	3 (2.1%)
Headache, n (%)	Door entrance of the MR/CT room	2 (2.7%)	4 (3.3%)	7 (4.8%)	3 (2.1%)
	Inside the MR/CT room	7 (9.3%)	2 (1.7%)	7 (4.8%)	6 (4.1%)
	Next to the gantry opening	1 (1.3%)	4 (3.3%)	0 (0%)	2 (1.4%)
	Sudden movement	1 (1.3%)	1 (0.8%)	1 (0.7%)	1 (0.7%)
	Turning head	2 (2.7%)	0 (0%)	1 (0.7%)	1 (0.7%)
	Leaning towards the gantry opening	1 (1.3%)	1 (0.8%)	0 (0%)	3 (2.1%)
Fatigue/drowsiness, n (%)	Door entrance of the MR/CT room	5 (6.7%)	5 (4.1%)	6 (4.1%)	6 (4.1%)
	Inside the MR/CT room	7 (9.3%)	3 (2.5%)	6 (4.1%)	4 (2.7%)
	Next to the gantry opening	1 (1.3%)	4 (3.3%)	0 (0%)	3 (2.1%)
	Sudden movement	0 (0%)	1 (0.8%)	1 (0.7%)	2 (1.4%)
	Turning head	0 (0%)	2 (1.7%)	0 (0%)	2 (1.4%)
	Leaning towards the gantry opening	0 (0%)	4 (3.3%)	0 (0%)	4 (2.7%)
**Acoustic noise**					
Tinnitus/ringing sound, n (%)	Door entrance of the MR/CT room	2 (2.7%)	3 (2.5%)	6 (4.1%)	2 (1.4%)
	Inside the MR/CT room	9 (12%)	3 (2.5%)	7 (4.8%)	4 (2.7%)
	Next to the gantry opening	3 (4%)	3 (2.5%)	2 (1.4%)	1 (0.7%)
	Sudden movement	0 (0%)	1 (0.8%)	1 (0.7%)	0 (0%)
	Turning head	1 (1.3%)	2 (1.7%)	1 (0.7%)	0 (0%)
	Leaning towards the gantry opening	0 (0%)	2 (1.7%)	0 (0%)	1 (0.7%)

Participants were asked that if a symptom is attributed during work at CT or MR, where are they located in relation to the CT or MR scanner, and what types of movement are they doing, if any. All questions allowed for multiple-choice alternatives, i.e., each participant could choose none, one, or multiple alternatives for each question. Therefore, % for each alternative is based on the maximum individual participants in each group (CT: 75; MR: 121; mixed: 146). Note that the mixed group answered each statement twice—once with regards to CT and once with regards to MR.

**Table 5:** Hearing and acoustic noise (*n* = 342)

		CT (n = 75)	MR (n = 121)	Mixed (n = 146)	
Self-rated hearing function	Normal, n (%)	55 (75%)	91 (81%)	106 (76%)	
	Impaired, n (%)	18 (25%)	21 (19%)	33 (24%)	
	Missing^a^, n (%)	2 (2.7%)	9 (7.4%)	7 (4.8%)	
Using hearing aid	No, n (%)	68 (96%)	111 (99%)	139 (100%)	
	Yes, n (%)	3 (4.2%)	1 (0.9%)	0 (0%)	
	Missing^a^, n (%)	4 (5.3%)	9 (7.4%)	7 (4.8%)	
Confirmed hearing loss	No, n (%)	64 (89%)	102 (92%)	126 (91%)	
	Yes, n (%)	8 (11%)	9 (8.1%)	13 (9.4%)	
	Missing^a^, n (%)	3 (4.0%)	10 (8.3%)	7 (4.8%)	
Are you experiencing acoustic noise at work in general as troublesome?	Not at all, n (%)	16 (23%)	36 (32%)	33 (24%)	
	Somewhat, n (%)	30 (42%)	56 (49%)	69 (51%)	
	A great deal, n (%)	25 (35%)	22 (19%)	34 (25%)	
	Missing^a^, n (%)	4 (5.3%)	7 (5.8%)	10 (6.8%)	
Are you experiencing acoustic noise at work at work in CT as troublesome?	Not at all, n (%)	15 (21%)	---	54 (41%)	
	Somewhat, n (%)	33 (45%)	---	51 (39%)	
	A great deal, n (%)	25 (34%)	---	26 (20%)	
	Missing^a^, n (%)	2 (2.7%)	---	15 (10%)	
Are you experiencing acoustic noise at work in MR as troublesome?	Not at all, n (%)	---	38 (33%)	41 (29%)	
	Somewhat, n (%)	---	61 (53%)	72 (52%)	
	A great deal, n (%)	---	16 (14%)	22 (16%)	
	Missing^a^, n (%)	---	6 (5.0%)	11 (7.5%)	
				At CT	At MR
Are you using hearing protection when you are inside the scanner room during imaging?	Always, n (%)	0 (0%)	77 (66%)	0 (0%)	93 (68%)
	Often, n (%)	0 (0%)	13 (11%)	0 (0%)	4 (2.9%)
	Sometimes, n (%)	0 (0%)	4 (3.4%)	0 (0%)	6 (4.4%)
	Never, n (%)	61 (84%)	5 (4.3%)	91 (69%)	7 (5.1%)
	I’m never in the scanner room during imaging, n (%)	12 (16%)	17 (15%)	40 (31%)	27 (20%)
	Missing^a^, n (%)	2 (2.7%)	5 (4.1%)	15 (10%)	9 (6.2%)
Is the sound-proofing sufficient in your control room or rooms? (Multiple choice) *Same for all CT or MR equipment*	Yes, n (%)	20 (27%)	38 (31%)	49 (34%)	64 (44%)
	Yes, noise measurements have been done, n (%)	4 (5.3%)	12 (9.9%)	7 (4.8%)	18 (12%)
	No, n (%)	3 (4.0%)	14 (12%)	8 (5.5%)	9 (6.2%)
	No, but noise measurements have been done, n (%)	2 (2.7%)	5 (4.1%)	4 (2.7%)	3 (2.1%)
	Sound-proofing has improved, n (%)	0 (0%)	4 (3.3%)	3 (2.1%)	4 (2.7%)
	I don’t know if noise measurements have been done, n (%)	24 (32%)	42 (35%)	51 (35%)	47 (32%)

Data are presented in counts and proportions (%). ^a^Missing data are accounted for by calculating % based on the number of responses for each item, except for the item “Is the sound-proofing sufficient in your control room or rooms?” (multiple choice) where % are based on maximum individual participants in each group. Differences in self-rated hearing function (*p* = 0.54) and established hearing loss (*p* = 0.79) were tested with the Chi-square test

The amount of weekly MR working hours (*n* = 529 participants) had no significant association with symptom prevalence among the *SMF*, *general work environment*, or *acoustic noise* symptom groups (all *p* > 0.05, data not shown). Instead, stress was revealed as a significant confounder to all symptom variables individually (all *p* < 0.05) except for metallic taste (*p* = 0.18), and within the three larger symptom groups (*SMF*, *p* < 0.001; *general work environment*, *p* < 0.001; and *acoustic noise*, *p* < 0.001). Additionally, for each year of age, the odds of experiencing ringing sensations or sound (tinnitus) one or more times a week increased by 3% (OR: 1.03; CI_95_: 1.00–1.06, *p* = 0.03). For unusual drowsiness, each year of life decreased the odds by 2.8% (OR: 0.97; CI_95_: 0.95–0.99, *p* = 0.01). Female sex increased the odds of headache 3.5-fold (OR: 3.47; CI_95_: 1.32–9.08, *p* = 0.01), while each year of life decreased odds of headache by 3.7% (OR 0.96; CI_95_: 0.94–0.99, *p* < 0.01). For sleeping difficulties, each year of life increased the odds by 3.1% (OR 1.03; CI_95_: 1.01–1.05, *p* < 0.01).

When looking exploratory at symptom occurrence (Table 3) and attribution (Table 4) in relation to being in the MR or CT room, vertigo was reported to occur “sometimes” by 49% in the MR group, and movement close to the MR gantry was the largest trigger (Table 3). In comparison, 16% of the CT group reported vertigo “sometimes” inside the CT room, most commonly when making sudden movements. Furthermore, MR personnel reported nausea, illusion of movement, and metallic taste to mostly occur when being close to, or leaning towards, the MR gantry (Table 4). Difficulties concentrating were reported to occur “sometimes” by 25% of CT personnel as compared to 14% by MR personnel (Table 3). However, we found no significant differences in general work environment symptoms between the MR, CT, and mixed groups (all *p* > 0.05) (Table 2).

**Fig. 2:**
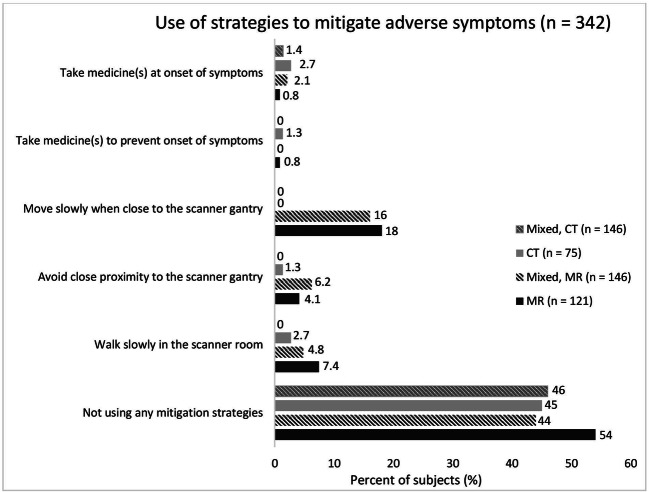
Percentages (%) of participants’ (CT personnel, MR personnel, and mixed personnel—the latter replied once with regards to CT and once with regards to MR) use of strategies to mitigate adverse symptoms during their work within CT and/or MR

Considering the use of strategies to mitigate adverse symptoms during work (Fig. 2), 54% and 45% among MR and CT personnel, respectively, reported no use of strategies. However, personnel in the MR group and the mixed group reported that they slowed down their movements in the MR scanner room (4.8–7.4%) and avoided working within proximity to the scanner gantry (16–18%), compared to the CT working personnel (0–2.7%). Regarding the use of hearing protection, 66–68% of the MR group and mixed group always used it inside the MR room during scanning, whereas 69–84% of the mixed group and CT group never used hearing protection inside the CT room during scanning (Table 5). Incidentally, two CT radiographers stated in an open question that they often closed the scanner room door to suppress acoustic noise even if no patient was examined.

## Discussion

The prevalence of reported adverse symptoms subjectively associated with SMF and acoustic noise exposure did not significantly differ between MR and CT radiographers. However, exposure to higher SMFs (3 T) increased MR radiographers’ odds of experiencing SMF related symptoms. Further, stress appears to have stronger associations with symptom prevalence than working with MRI.

Although this study partly corroborates previous literature [3, 8, 13, 23, 28–30], our data show no significant association between SMF symptoms and the amount of weekly MR working hours. In contrast, de Vocht et al [13] did see a positive association between weekly MR working hours and increased reporting of SMF symptoms; however, their data failed to associate subjective symptoms with magnetic field exposure or magnetic field strength (1.5 T and 3 T). In comparison, Schaap et al [3] found a positive association between increased SMF symptoms and increased field strength in closed-bore MR systems. Unlike their study, however, our material had a nearly complete absence of 7 T personnel. Although not significant, we found tendencies towards increased SMF symptom occurrence and attribution among MR personnel considering reported vertigo, nausea, metallic taste, and illusion of movement compared to CT personnel. These symptoms were mainly reported when participants worked close to the scanner bore and during movement, i.e. during exposure to strong SMFs and increased motion-induced time-varying magnetic fields. Given that the demand for stronger field MR systems keeps growing, we might encounter higher symptom prevalence in the future [31]. Considering the inconsistent results, radiology departments need to establish education and protective measures to minimize the risk of SMF-related adverse health effects for MR personnel.

Another key finding in our study was the influence of stress on reported symptoms, regardless of image modality. As our study did not delve any deeper into organizational structures or work culture, nor aimed to evaluate environmental stressors, we cannot establish any causality or pinpoint specific stress-related objectives to be addressed for decreasing symptom occurrence. However, other studies have also found stress to be an important contributing factor in reported health effects by MR personnel [13, 30]. Working shifts, working in larger departments, and/or having 6–15 years of work experience were the greatest stressors among Finnish radiographers [32]. Conversely, stress was not seen to impact the symptom prevalence in general among MR personnel in a previous Swedish study [8]. In that study, however, no control group was included and the number of participants was limited (*n* = 59).

Head ringing/tinnitus has been reported during SMF exposure [3, 29, 33]. We chose to look at head ringing separately from all other symptoms because it also can be triggered by acoustic noise [34]. Accordingly, CT personnel reported head ringing when being inside the CT room. Moreover, CT personnel revealed a greater risk of experiencing acoustic noise in their work environment as troublesome. While we lack data on sound pressure levels and on noise characteristics that were perceived as troublesome, we hypothesize from clinical experience that fans and X-ray tube rotations contribute to noise nuisance. Increased patient and personnel turnover during CT shifts might also aggravate noise exposure. On the other hand, awareness of acoustic noise and sound pressure levels from MR scanners, at least in Sweden, has led to site building revisions regarding acoustics in MR workspaces—but not to the same extent regarding CT environments.

One explanation for the MR radiographers’ mildly reported health complaints in our study might be due to adaptation to the work environment. The adaptation theory is supported by comparing the symptom outcome among MR personnel with less than one year of experience, as done by Zanotti et al [23, 35]. There, participants reported symptoms more frequently during their first two months at MR, but the prevalence declined thereafter. Incidentally, the mean work experience with MR was rather high in our study cohort (14 years). Experienced MR personnel have likely developed habits to mitigate adverse effects from both SMF and acoustic noise exposure, such as moving slowly within the SMF vicinity and wearing hearing protection during scanning. Additionally, the so-called healthy worker effect [36] cannot be ruled out as a bias among MR personnel in our study, since the strong magnetic fields make it problematic for certain personnel to enter the scanning room, i.e., persons with active implants, or those who experience severe symptoms in general. However, our outcome might also reflect a successful result of the 2013 implemented EU guidelines [22]—which acknowledge MR as an occupational risk environment and states both exposure limits and strategies to minimize adverse health effects. The CT environment outside of radiation safety may have been foreshadowed in that regard.

## Limitations

Any subjectively reported hearing function should be interpreted with caution as participant-perceived ratings may lack correlation with audiometric data, and hearing impairment is often underreported [37]. Audiometric testing would, however, not have been feasible given our study design. Participants’ experience of work environmental noise and adaptation to mitigation strategies thereof still highlighted acoustic noise as an issue in MR and CT work environments.

Our way of distributing the questionnaires left us no possibility to control how many workers had been asked to partake in the study. A brief overview of the MR personnel in Sweden, however, gives us an approximate response rate of 60% [25]. Moreover, as our primary aim was to explore symptom reports from MR users, we wanted our controls (CT) to work within the same hospitals. Therefore, we chose not to send out the questionnaire to hospitals that did not have access to MRI. As such, our CT controls are less numerically representative compared to the MR cohort with regards to national demographics, which might induce a bias. Many of our participants were also working with other imaging modalities in addition to MR and/or CT, e.g., conventional radiography, which admittedly reflects normal working conditions for MR and CT personnel in Sweden. Still, such work environmental factors or organizational biases cannot be excluded as confounders, and might, conversely, be an explanation as to why adverse symptoms are not more prevalent in our study.

## Conclusion

Comparing MR personnel to a CT control group suggests that adverse symptoms cannot solely be explained by SMF exposure and acoustic noise exposure, and our hypothesis that MR radiographers report more symptoms in general than CT radiographers was rejected. Stress was an important confounder for symptom onset. However, SMF symptoms were significantly more prevalent among MR radiographers whose work included 3-T systems compared to those only working at ≤ 1.5 T. MR personnel also tended to use more mitigation strategies when working close to the scanner than did CT personnel. Acoustic noise was generally mitigated by MR personnel, whereas CT personnel experienced work environmental noise as more troublesome. This finding might imply that reducing noise nuisance within CT environments is needed and ought to be further looked into.

## Abbreviations

dB: Decibel
ICNIRP: International Commission on Non-Ionizing Radiation Protection
OR: Odds ratio
SMF: Static magnetic field
T: Tesla

## Declarations

### Guarantor

The scientific guarantor of this publication is Isabella M. Björkman-Burtscher (Department of Radiology, Institute of Clinical Sciences, Sahlgrenska Academy, University of Gothenburg, Gothenburg, Sweden; Department of Radiology, Sahlgrenska University Hospital, Västra Götalands Region, Gothenburg, Sweden).

### Statistics and biometry

No complex statistical methods were necessary for this paper.

### Study subjects or cohorts overlap

Some study subjects or cohorts have been previously reported in: Hansson B, Olsrud J, Wilén J, Owman T, Höglund P, Björkman-Burtscher IM (2020) Swedish national survey on MR safety compared with CT: a false sense of security? *Eur Radiol* 30:1918-1926.

### Methodology

• prospective

• cross-sectional study

• single-center study with a national study cohort recruitment
